# Hormonal Regulation of Oligodendrogenesis II: Implications for Myelin Repair

**DOI:** 10.3390/biom11020290

**Published:** 2021-02-16

**Authors:** Jocelyn M. Breton, Kimberly L. P. Long, Matthew K. Barraza, Olga S. Perloff, Daniela Kaufer

**Affiliations:** 1 Helen Wills Neuroscience Institute, University of California Berkeley, Berkeley, CA 94720, USA; kimberly.long@ucsf.edu (K.L.P.L.); danielak@berkeley.edu (D.K.); 2 Molecular and Cellular Biology, University of California Berkeley, Berkeley, CA 94720, USA; mbarraza@berkeley.edu; 3 Memory and Aging Center, Department of Neurology, University of California San Francisco, San Francisco, CA 94143, USA; olga.litvin@ucsf.edu; 4 Integrative Biology, University of California Berkeley, Berkeley, CA 94720, USA; 5 Canadian Institute for Advanced Research, Toronto, ON M5G1M1, Canada

**Keywords:** oligodendrogenesis, remyelination, hormones, steroids, peptides

## Abstract

Alterations in myelin, the protective and insulating sheath surrounding axons, affect brain function, as is evident in demyelinating diseases where the loss of myelin leads to cognitive and motor dysfunction. Recent evidence suggests that changes in myelination, including both hyper- and hypo-myelination, may also play a role in numerous neurological and psychiatric diseases. Protecting myelin and promoting remyelination is thus crucial for a wide range of disorders. Oligodendrocytes (OLs) are the cells that generate myelin, and oligodendrogenesis, the creation of new OLs, continues throughout life and is necessary for myelin plasticity and remyelination. Understanding the regulation of oligodendrogenesis and myelin plasticity within disease contexts is, therefore, critical for the development of novel therapeutic targets. In our companion manuscript, we review literature demonstrating that multiple hormone classes are involved in the regulation of oligodendrogenesis under physiological conditions. The majority of hormones enhance oligodendrogenesis, increasing oligodendrocyte precursor cell differentiation and inducing maturation and myelin production in OLs. Thus, hormonal treatments present a promising route to promote remyelination. Here, we review the literature on hormonal regulation of oligodendrogenesis within the context of disorders. We focus on steroid hormones, including glucocorticoids and sex hormones, peptide hormones such as insulin-like growth factor 1, and thyroid hormones. For each hormone, we describe whether they aid in OL survival, differentiation, or remyelination, and we discuss their mechanisms of action, if known. Several of these hormones have yielded promising results in both animal models and in human conditions; however, a better understanding of hormonal effects, interactions, and their mechanisms will ultimately lead to more targeted therapeutics for myelin repair.

## 1. Introduction

Many neurological disorders are characterized by changes in myelin, the protective and insulating sheath surrounding axons. Such disorders include psychiatric disorders, Alzheimer’s Disease (AD), and demyelinating disorders, conditions in which myelin is damaged and ultimately lost [1,2,3,4]. In addition to its canonical role in saltatory conduction, myelin has been increasingly implicated in a wide range of central nervous system (CNS) functions, such as neural synchrony, synaptic function, and trophic support of myelinated axons [5,6,7]. As a result, extensive myelin loss can be accompanied by a host of secondary pathologies that lead to deficits in cognition and motor function [8,9,10]. Disorders characterized by white matter loss, including demyelinating disorders, are highly prevalent, with multiple sclerosis (MS) alone affecting more than one in 500 individuals [11]. Currently, there are no cures and few therapeutic options for demyelinating conditions. Thus, understanding the mechanisms to preserve myelin and promote remyelination is critical to the development of new and effective treatments.

**Figure 1:**
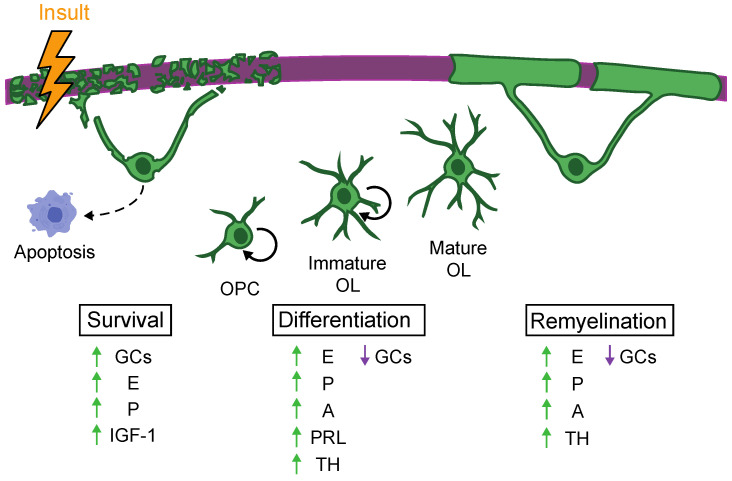
After an insult that leads to myelin degradation, oligodendrocyte (OL) survival, oligodendrogenesis, and remyelination are differentially affected by various classes of hormones. Green arrow = promote, Purple arrow = downregulate. GCs = glucocorticoids, E = estrogens, P = progestogens, A = androgens, IGF-1 = insulin-like growth factor-1, PRL = prolactin, TH = thyroid hormones.

Oligodendrocytes (OLs) are the glial cells in the CNS that generate the myelin sheath. Once matured, these cells may have a dramatically limited capacity for creating new myelin [12,13,14]. In addition, injury, inflammation, and myelin damage can lead to widespread OL cell death [15,16,17,18]. As a result, oligodendrogenesis, the creation of new OLs, is crucial for remyelination following injury (Figure 1) [19,20,21]. However, remyelination is often impaired in demyelinating disorders, which may be due to deficits in oligodendrogenesis [22]. For example, oligodendrocyte precursor cells (OPCs) are compromised in their ability to differentiate into mature, myelinating OLs in MS patients [23]. Understanding the mechanisms of both normal and altered oligodendrogenesis and subsequent myelination is important not only for disorders such as MS but also for all disorders characterized by changes in CNS myelination, including spinal cord injury (SCI), stroke, AD, post-traumatic stress disorder (PTSD), and other psychiatric disorders [1,3,4,24,25,26].

The mechanisms underlying the process of remyelination can be investigated in animal models that replicate some aspects of human pathologies. The most commonly used animal model for MS is experimental autoimmune encephalomyelitis (EAE), which involves the induction of an immune response to myelin antigens. This leads to the progressive loss of myelin, as well as cognitive and motor deficits [27,28]. In addition, acute or chronic demyelination can be triggered by toxins such as lysophosphatidylcholine (LPC) and cuprizone, which induce local inflammation or directly induce OL apoptosis, respectively [29,30]. Lastly, myelin loss can be induced by physical injury (as observed in SCI) and hypoxia (either induced directly or indirectly as a result of ischemia) [31,32]. Collectively, these animal models allow us to test, both in vitro and in vivo, the factors and cellular mechanisms that may progress myelin pathology or that, conversely, may aid in myelin repair.

Oligodendrogenesis is regulated by numerous endogenous factors, including many different hormones (for a detailed review, see Reference [33]). In fact, MS, the most common demyelinating disorder, is almost three times more common in women than in men, suggesting that sex hormones may play a role in disease pathology [11]. In addition, women with MS often have fewer relapses during pregnancy, at a time when sex hormones and peptide hormones like prolactin are at their peak [34]. Many hormones, including insulin-like growth factor 1 (IGF-1), thyroid hormones (THs), and steroid hormones, also influence oligodendrogenesis during development and throughout adulthood. Hence, understanding hormonal effects on oligodendrogenesis and remyelination in models of myelin pathology provides an avenue for ultimately improving myelin repair.

In this review, we will explore how hormonal factors affect oligodendrogenesis within disease contexts. The accompanying review introduces the hormones discussed and explores the hormonal regulation of oligodendrogenesis under physiological conditions [33]. As in our companion review, we will restrict our discussion to the “classic” endocrine signaling molecules, which are typically released from a gland into circulation to act upon distant tissues. We will describe the effects of classic steroid hormones, peptide hormones, and THs. For each category, we will note the hormone’s effects on animal models characterized by impaired myelination, and we will discuss their relevance to findings in human disorders. While most of the literature we review is focused on demyelinating disorders, we also note relevance for additional diseases. We end with a discussion of future directions. Overall, we advocate for more extensive research into hormones as a potential therapeutic target for myelin repair.

## 2. Steroid Hormones

Steroids are hydrophobic molecules synthesized from cholesterol that have important actions within the CNS, including regulation of oligodendrogenesis [33]. For this review, we will focus on a subset of steroid hormones synthesized primarily in the adrenal cortex and gonads, namely, glucocorticoids (GCs) and sex hormones, which canonically act through nuclear receptor signaling [35,36]. As we describe in our companion review, steroid hormones act to increase OPC differentiation and enhance maturation/myelination of OLs under physiological conditions [33]. In line with their effects on oligodendrogenesis, stress hormones such as GCs, and sex hormones such as estrogens, progestogens, and androgens, are associated with MS and other myelin related diseases and may aid in some aspects of myelin repair [37,38,39].

### 2.1. Glucocorticoids (GCs)

GCs are one of the primary stress hormones. This family includes endogenous cortisol (the primary GC for humans), corticosterone (the primary GC for rodents), and synthetic hormones such as dexamethasone (Dex). Like many steroid hormones, GCs enhance oligodendrogenesis in vitro and in vivo, specifically by enhancing differentiation of neural stem cells (NSCs) and OPCs, via activation of glucocorticoid receptors (GRs) [33,40], and ultimately increasing expression of myelin basic protein (MBP) [41,42]. However, prolonged exposure to corticosterone or Dex can reduce myelination, indicating the effects of GCs may depend on dosing and duration [43]. It is important to note that in disease states, particularly in autoimmune diseases such as MS, GCs can affect myelination either by directly activating GRs in OPCs and OLs to modulate proliferation, differentiation, survival, or myelination rate, or indirectly through modulation of immune functions.

#### Implications for Disorders

Levels of circulating GC hormones can be increased by stress, injury, and disease, and although prolonged or high levels of GCs may be harmful, acute increases in GCs may in fact be beneficial. For example, methylprednisolone (MP), a synthetic GR agonist, often given to spinal cord patients within a few hours of injury, protects against OL cell death following SCI in mice; specifically, MP treatment after SCI increases the number of mature OLs at the site of injury eight days later [44]. This protective effect was unique to OLs and was dependent on GR signaling [44]. In a similar study, MP was found to protect against α-amino-3-hydroxy-5-methyl-4-isoxazolepropionic acid (AMPA)-induced excitotoxicity, an effect that was causally related to its upregulation of the neuroprotective cytokine erythropoietin [45]. In addition to effects on OL survival, MP also affects OPC proliferation. Specifically, MP treatment reduced the number of proliferating neural progenitor cells and OPCs labeled one to six days after SCI. This effect was only observed in the short term; MP did not affect OPC proliferation one month after injury [46]. While these findings call into question whether MP is beneficial for myelin repair, this study did not address whether a reduction in OPCs was due to increased differentiation into mature OLs, which would in fact aid in injury recovery.

OL apoptosis, often induced by inflammation, is important in MS as well as other demyelinating disorders [8,47,48]. In animal models of demyelination, GCs are protective against OL and myelin loss by preventing inflammatory cytokine-induced OL apoptosis. Specifically, the synthetic GC prednisone, which is often given to MS patients for its anti-inflammatory properties, alleviates cuprizone-induced demyelination and inhibits inflammatory cytokines and signaling pathways in mice [49]. Furthermore, GCs protect OPCs and OLs from pro-inflammatory cytokine-induced cell death [50,51]. Future work should aim to determine if GC protection from cell death is mediated by immunosuppressive effects or directly action on OLs.

Despite this evidence for prevention of OL loss, there is conflicting evidence of whether GCs can enhance remyelination in an injury or disease context. While GCs such as Dex and MP accelerate OPC differentiation in culture, GCs impair remyelination in the corpus callosum in vivo in cuprizone-treated mice [52]. Thus, while GCs can push OLs to mature, this enhanced maturation does not always correspond with enhanced myelination. More research is needed to test how GCs affect OL development and myelination, particularly in these injury and disease models. In addition, GCs act in concert with other hormones in vivo, and such interactions may drive different outcomes of oligodendrogenesis and myelination. For example, Dex down-regulates the expression of IGF-1, the IGF-1 receptor, and IGF-1 binding proteins [53]. Thus, exposure to high levels of GCs could impair the action of otherwise pro-oligodendrogenesis hormones like IGF-1, thereby indirectly inhibiting oligodendrogenesis. Such hormonal interactions will be important to consider in disease models in vivo.

Understanding how GCs affect oligodendrogenesis has important broader implications not only for demyelinating disorders but also for human disorders characterized by alterations in the hypothalamic–pituitary–adrenal axis and changes in cortisol, including PTSD and depression [54,55]. Interestingly, these disorders are associated with changes in myelin [25,56] (see Box 1), and GCs may provide one mechanism by which these alterations arise. For example, in patients with major depressive disorder, elevated cortisol is correlated with reduced white matter integrity in fronto-subcortical and fronto-limbic systems [57]. In our own work using animal models, we recently identified sex-, age-, and region-specific changes in OLs and myelin following exposure to acute trauma. Juvenile exposure to acute stress led to long-lasting reductions in grey matter myelin in female, but not male, adult rats [58]. In addition, male rats demonstrated short-term changes in myelin content; these changes were associated with corticosterone levels during stress exposure [58]. Furthermore, in adult male rats exposed to the same acute stressor, hippocampal and amygdala myelin levels positively correlated with avoidance and fear scores, respectively [59]. More research is needed to identify a causal role for GCs in altering OLs and myelin in stress-associated disorders.

Box 1 **Alterations of myelin in stress-related neuropsychiatric disorders [60,61,62,63,64,65,66,67,68,69,70,71,72].**

Many neuropsychiatric disorders are characterized by alterations in myelin, both in white matter and grey matter regions [1,25,60]. White matter myelin is composed primarily of bundles of myelinated axons, while grey matter myelin is less dense, with myelinated axons closer to cell bodies and dendrites [61]. Alterations in myelin have been implicated specifically in a number of stress-related mental health disorders, including depression and PTSD, suggesting myelin, and the OLs that generate it, might play a functional role in mood [25,60,62,63,64,65,66,67]. For example, depression patients demonstrate reduced white matter integrity and intensity across many brain regions, especially in areas such as the prefrontal cortex [60,65,67,68]. These alterations in myelin may occur through decreases in OL density, altered expression of OL related genes, and/or changes in OL morphology, all of which have been observed in depression [60,69]. Intriguingly, changes in white matter in the fronto-limbic system may correlate with behavioral symptoms of depression such as rumination [70]. Alterations in white matter myelin are also observed in PTSD patients, with reductions in white matter volume in many areas, yet increases in others, highlighting the regional heterogeneity of trauma’s impact on myelin [71]. In addition to changes in white matter, PTSD patients demonstrate alterations in grey matter myelin; veterans with PTSD have increased hippocampal grey matter myelin content compared to trauma-exposed controls and interestingly, this increase in hippocampal myelin positively correlates with PTSD symptom severity [25]. In a recent study, changes in white matter myelin were also found to positively correlate with PTSD symptoms [72]. Altogether, more work will be needed to piece apart regional changes in white matter and grey matter myelin in these stress-induced disorders, as well as the underlying mechanisms by which alterations in myelin arise. Finally, the studies we describe here only scratch the surface of a rich field of literature.

### 2.2. Sex Hormones

**Figure 2:**
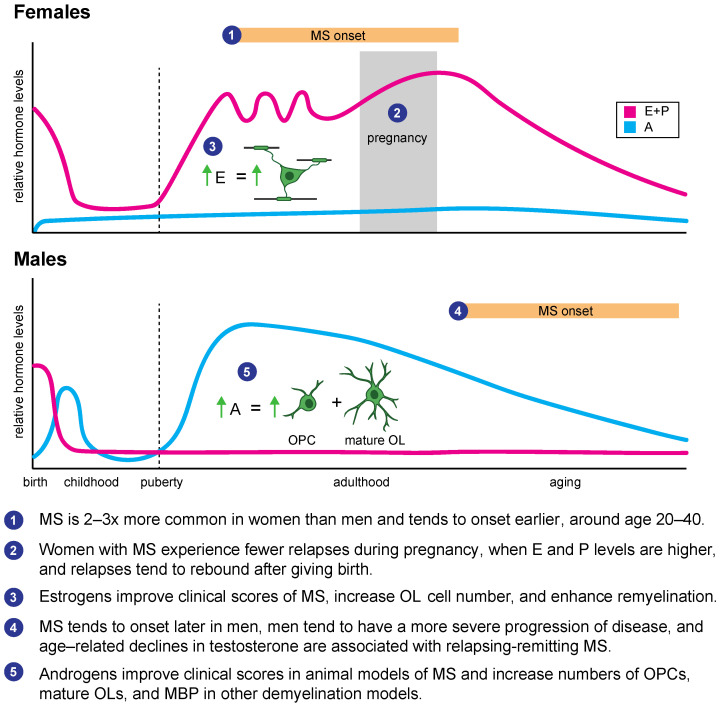
Summary of sex hormone changes across development and their relationship with demyelinating disorders, oligodendrogenesis, and remyelination. Dashed line = puberty onset. E = estrogens, P = progestogens, A = androgens.

Sex hormones, including the estrogens, progestogens, and androgens, all modulate oligodendrogenesis and myelogenesis, as we describe in our companion review [33,73,74]. Indeed, males and females are differentially affected by demyelinating disorders such as MS [11], and differences in sex hormones might account for some of this sex-specific risk (Figure 2).

#### 2.2.1. Estrogens

Estrogens, the major family of female sex hormones, are produced primarily by the ovaries. The most potent estrogen, 17-β estradiol (E2), has many physiological functions for both male and female animals, including regulation of oligodendrogenesis [33]. Broadly, E2 promotes OPC differentiation and OL maturation, acting through its two nuclear estrogen receptors (ERs), ERɑ and ERβ, and through its membrane-bound receptor GPR30, all of which are expressed by OLs [75,76,77,78,79,80].

##### Implications for Disorders

Estrogen treatments may have protective and rehabilitative properties for OLs, with important implications for demyelinating disorders. Indeed, estrogen treatments improve clinical outcomes for MS patients and are now ready for phase three clinical trials [81,82,83]. In animal models, E2 protects against hypoxia/ischemia and SCI-induced OL cell death and white matter damage [84,85]. Estrogens also promote remyelination and reduce the loss of OLs in MS models and following cuprizone-induced demyelination [86,87]. For example, in an animal model of EAE, an E2 agonist increased both the number of OLs as well as axon myelination [88]. Thus, estrogens aid in OL survival and promote remyelination following injury. These protective and pro-myelinating effects may occur through several ER-mediated mechanisms. In immature and mature OLs, E2 decreases the cytotoxic effect of free radical donors, which are implicated in OL damage and MS pathology [89,90]; this protective effect was blocked by an ER antagonist [79]. Binding of ERβ also activates the phosphatidylinositol 3-kinase/protein kinase B/mammalian target of rapamycin signaling pathway in OLs, thereby promoting OL survival and axon remyelination [91]. Activation of the membrane-bound GPR30 receptor also contributes to improved remyelination in cuprizone-induced demyelination models [92]. In both the spinal cord and in the corpus callosum, a specific GPR30 agonist elicits both increased OPC proliferation and maturation, prompting OPCs to develop into immature OLs. Further, animals treated with GPR30 agonists showed increased MBP immunoreactivity and thicker axon diameters [92].

In addition to ER-mediated effects, estrogens may aid remyelination, in part, via interactions with other hormones. For example, in cuprizone-induced demyelination models, E2 induces production of IGF-1 by astrocytes, which in turn promotes OL proliferation and differentiation [93]. In addition, estradiol increases progesterone receptor (PR) expression in OL cultures [94], and progesterone, as described in our companion review, promotes oligodendrogenesis [33]. Combining estradiol with progesterone enhances remyelination to a greater extent than either hormone alone and increases the number of immature and mature OLs following cuprizone-induced demyelination in mice [93]. Furthermore, a combination of these hormones reduces infiltration of inflammatory cells in EAE mouse models [38]. Thus, administration of both progesterone and estradiol produces synergistic effects, more effectively restoring myelination and reducing inflammation [38,93]. Collectively, these findings emphasize the need to investigate the complex interactions between hormones in the context of disorders.

Estrogen replacement therapy may also play a role in protecting middle-aged women from adverse effects following menopause, including myelin abnormalities and impaired cognition [95,96,97]. Middle-aged (nine to 12 month old) female ovariectomized (OVX) rats receiving one month of E2 replacement therapy retained a higher volume of white matter myelin sheaths compared to OVX rats receiving placebo [96,98]. Myelin fiber length and diameter increased, which correlated with improved spatial learning in the OVX middle-aged female rats [96]. Thus, estrogen replacement therapy’s benefits on cognitive decline may arise, in part, via protection of white matter [99]. The mechanisms of estrogen’s beneficial effects remain to be determined, and indeed, future work may determine if this protection occurs via OL survival and/or effects on oligodendrogenesis.

#### 2.2.2. Progestogens

The steroid hormone progesterone is commonly known for its role in the maintenance of pregnancy, yet it also has a wide range of functions in the body and throughout the CNS, including effects on oligodendrogenesis [33]. Progesterone acts primarily on nuclear progesterone receptors (PRs) to stimulate OPC differentiation and upregulate MBP levels [100]. Progesterone can also act via membrane-bound PR, and interestingly membrane-bound PRs, though typically only found in neurons, are expressed in OLs following traumatic brain injury, suggesting they may play a selective role in injury recovery [101].

##### Implications for Disorders

Progesterone has a significant impact on remyelination (for a recent review, see Reference [73]). Progesterone’s ability to repair myelin in the adult CNS extends across multiple injury models, including SCI, LPC or cuprizone-induced demyelination, and EAE.

In SCI models, chronic treatments with progesterone enhance OPC proliferation, differentiation, and remyelination following injury [102,103,104,105,106,107]. In fact, OPC differentiation is arrested after SCI and is reinstated following treatments with progesterone [104]. While a complete mechanism for this effect has not been determined, progesterone may exert indirect effects on OL differentiation through upregulation of transforming growth factor-beta 1, a known OL differentiation factor, in microglia and astrocytes in the spinal cord [102]. In addition to effects on differentiation and remyelination, progesterone also improves the survival of OPCs following injury, in part by reducing levels of pro-inflammatory cytokines [108]. This effect requires a functional PR and is not observed in PR knock-out animals [108]. Lastly, progesterone-induced increases in mature OLs and MBP immunoreactivity are associated with positive functional outcomes, such as improved gait, making progesterone a promising future avenue for treatment [107].

Similar to findings in SCI models, progesterone has protective and pro-remyelinating effects in LPC, cuprizone, and other toxin-induced demyelination models [109,110,111]. Progesterone not only enhances myelination of axons, it also increases the density of OPCs and mature OLs [109]. Progesterone acts to increase OPC proliferation, differentiation, and migration to the injury site via a mechanism involving the nuclear PRs [110]. Furthermore, nestorone, a synthetic derivative of progesterone that selectively targets PR, stimulates OPC proliferation, migration, and differentiation in cerebellar slice cultures treated with LPC at a much lower dose than natural progesterone [110]. Similar findings are observed in vivo, where progesterone and nestorone increase the density of OPCs and mature OLs, and enhance the formation of myelin proteins such as PLP and MBP in cuprizone demyelination models [93,112]. Again, these effects were dependent on PR and were not observed in PR knock-out animals [112].

In the rodent EAE model, progesterone may improve remyelination through beneficial influences on inflammation, specifically by reducing inflammatory cell infiltration, proinflammatory cytokine levels, and numbers of reactive microglia [38,113,114,115]. In addition, progesterone affects a transcription factor that contributes to OL differentiation, oligodendrocyte transcription factor 1 (Olig1), increasing its movement from the cytoplasm into the nucleus and promoting OPC differentiation [116]. Functionally, treatment of EAE animals with progesterone improves clinical outcomes [113,115,117]. Together, these findings position progesterone as a strong candidate for future therapies in MS patients. Interestingly, MS patients express lower levels of the progesterone metabolite, allopregnanolone [39]. Reduced allopregnanolone may contribute to the impaired OPC differentiation observed in MS. However, whether reduced neurosteroid synthesis and metabolism contribute to disease pathology or are simply biomarkers remains unknown.

While progesterone has primarily been studied in the context of SCI and demyelinating disorders, it also has beneficial effects for other disorders associated with myelin loss, such as AD and stroke [3,118,119]. For example, progesterone and allopregnanolone have protective effects in an animal model of AD, increasing not only neurogenesis but also the expression of 2’,3’-Cyclic-nucleotide 3’-phosphodiesterase (CNPase), a myelin associated enzyme that marks mature OLs [120,121]. Thus, progesterone may have protective effects across the lifespan. Furthermore, following a stroke, progesterone promotes increased density of both OPCs and mature OLs [122]. Altogether, progesterone shows the same pro-oligodendrogenesis effects in adult animals following injury as that observed in development [33]. Future work will undoubtedly continue to explore the mechanisms by which progesterone acts and to assess the functional impact of oligodendrogenesis in these models; in particular, little is known about the role of membrane-bound PRs in demyelinating disorders.

#### 2.2.3. Androgens

Androgens are a class of steroid hormones that includes testosterone (the primary circulating androgen in males), dihydrotestosterone (DHT, a metabolite of testosterone and the most potent androgen), and several weakly acting hormones [123]. Androgens primarily act via a nuclear receptor, the androgen receptor (AR). While it remains unclear whether androgens act directly on OLs, androgen signaling through the AR promotes oligodendrogenesis and subsequent myelination in vivo, though other in vitro work suggests that androgens may also moderately enhance OL cell death [33,124].

##### Implications for Disorders

Studies reporting androgen-induced OL cell death under physiological conditions are somewhat at odds with several studies, demonstrating a protective effect of testosterone in demyelinating disorders. Indeed, early studies suggested that in mouse EAE models, castration induces clinical relapses and a greater influx of activated T-cells into the CNS, suggesting that male gonadal hormones are protective [125]. Moreover, administration of testosterone prior to and concurrently with EAE induction results in reduced clinical scores in male and female mice, as well as increased expression of IL-10, an anti-inflammatory cytokine [126,127]. Administration of DHT, which cannot be aromatized to estrogens, yields similar improvements in clinical scores and neuroinflammatory markers in both mice and rats [127,128]. Given that androgens are known modulators of immune function, it is possible that these benefits in the EAE model may be attributed to the suppression of neuroinflammation rather than direct effects on OLs and myelin [129].

Androgens can also enhance remyelination following toxin-induced demyelination. For example, 12 weeks of cuprizone administration results in long-lasting loss of OLs and myelin in the corpus callosum of both castrated male and ovariectomized female mice; however, administering testosterone for six weeks following cuprizone withdrawal increases the number of OPCs and restores mature OL numbers and MBP expression in both sexes, suggesting that testosterone enhances OPC recruitment, differentiation, and remyelination [130]. This rescue is blocked by genetic knockout of AR function in neurons and macroglia (AR^Nestin-Cre^ mice). Furthermore, this myelin-rescuing effect can be mimicked in vitro in cerebellar slice culture; applying testosterone or DHT following LPC-induced demyelination restores myelination, an effect that is blocked by an AR antagonist. Together, these data suggest AR signaling has a direct role in CNS remyelination [130]. Similar results were found in a mouse model of spinal cord demyelination induced by LPC injection [131]. Specifically, three days of testosterone administered immediately following LPC injection increased myelination of the spinal cord. Notably, however, genetic knockout of CNS AR signaling did not completely block the effect of testosterone in this model, suggesting that, at least in the spinal cord, testosterone exerts some protective and/or remyelinating effects through non-AR signaling [131]. It was further demonstrated that testosterone increases the number of proliferating OL lineage cells in the LPC-treated spinal cord in vivo, and that testosterone increases the number of mature OLs in neonatal mouse-derived mixed glial cultures following LPC treatment in vitro [131]. Overall, these studies suggest that testosterone promotes remyelination by promoting OPC proliferation and differentiation and subsequent remyelination. Nonetheless, the exact mechanism of this rescue is unresolved. In particular, OL survival was not assessed in these experiments. In addition, it is unclear whether androgens act directly on OPCs and OLs, or indirectly through effects on surrounding cell types.

Although the mechanism of action remains somewhat unclear, the ability of androgens to protect against demyelination and enhance remyelination may ultimately have implications for disorders such as MS. Reports suggest that women are more susceptible to MS than men [11]; however, men may have a more aggressive progression of disease [132]. In addition, men tend to display a later onset of disease that coincides with age-related declines in testosterone levels, and lower testosterone levels are associated with greater disability scores and worse disease course in men with relapsing–remitting MS [133]. Androgens have, thus, emerged as a potential therapeutic target for MS. In a small preliminary clinical trial conducted in a cohort of 10 men with relapsing–remitting MS, testosterone supplementation enhanced performance in an auditory processing task and attenuated brain atrophy [134]. However, overall disability scores and lesion volumes were unaffected. Subsequent follow-up and analysis of this same cohort revealed that testosterone supplementation reduced, and perhaps even reversed, gray matter volume loss and promoted anti-inflammatory immune profiles [135,136]. Together with the animal literature, this may suggest that androgens inhibit neuroinflammation, which in turn attenuates tissue loss, but may have little effect on myelin lesions themselves. Understanding the direct and indirect effects of androgens on OLs will, therefore, be critical to the future of androgen therapy in demyelinating disorders. Larger, well-controlled clinical trials will enhance our understanding of androgens’ modulation of oligodendrogenesis in adulthood and their capacity to serve as therapeutic targets for demyelinating disorders. In sum, androgens offer a promising target to promote remyelination, but many questions remain surrounding the effects of androgens on oligodendrogenesis and remyelination. Namely, it is unclear whether androgens promote OL survival, whether they modulate oligodendrogenesis and remyelination outside of mouse models, and whether they act directly or indirectly on OLs.

## 3. Amino Acid-Based Hormones (Peptides, Amines, Thyroid Hormones)

In this section, we discuss the role of amino acid-derived hormones and their receptors in oligodendrogenesis and myelin repair. These hormones can be genetically encoded chains of two or more amino acids (peptides) or enzymatically altered compounds derived from single amino acids (amines and THs). As we describe in our companion review, many of these hormones act to enhance OPC proliferation and/or OL survival through common signaling pathways [33].

### 3.1. Insulin-Like Growth Factor 1 (IGF-1)

IGF-1 is a peptide that broadly contributes to cell growth, proliferation, differentiation, and survival. IGF-1 exerts its effects in part by binding the IGF-1 receptor (IGF1R), which is expressed in all CNS cell types, including OLs [137,138,139,140]. In a developmental context and under physiological conditions, IGF-1 promotes oligodendrogenesis and increases the number of mature, myelinating OLs by promoting OPC and OL survival and enhancing NSC and OPC differentiation [33,137,141].

#### Implications for Disorders

IGF-1 exerts protective effects in several animal models of myelin damage. In alignment with the anti-apoptotic effects of IGF-1 under physiological conditions, administering IGF-1 or constitutively overexpressing IGF-1 prevents OL apoptosis and/or myelin loss induced by a host of demyelinating conditions, including cuprizone treatment, LPC treatment, undernourishment, and ischemia [142,143,144,145]. An IGF1R agonist has similar protective effects in a mouse ischemia model [146]. Furthermore, in mouse mixed glial cultures, IGF-1 attenuates apoptosis of mature OLs induced by the inflammatory cytokine tumor necrosis factor alpha [147]. Notably, each of these studies administered IGF-1 immediately following myelin insult or utilized an animal model with constitutive overexpression of IGF-1.

IGF-1 has also been targeted as a potential treatment for demyelinating disorders such as MS. Interestingly, constitutive loss of IGF1R impairs OPC survival, proliferation, and subsequent OL remyelination after cuprizone-induced demyelination [138], again suggesting that IGF-1 plays an important role in OL survival. In the rodent EAE model, eight days or 10 days of IGF-1 administration improved clinical movement deficits and lesion numbers [148,149]. Despite these promising studies, however, other experiments call into question the efficacy of IGF-1 treatment. For example, while a 14-day treatment with IGF-1 ameliorated lesion severity in mice, this effect was transient, and IGF-1 treatment had no lasting effect on remyelination [150]. Furthermore, IGF-1 only conferred benefits when administered immediately following EAE induction; IGF-1 treatment that was begun well past clinical onset had no effects on disease severity and myelin lesions [150]. Similarly, viral-induced upregulation of IGF-1 begun eight days after LPC-induced demyelination in the spinal cord had no effect on remyelination in aged rats [151]. Together, these results may suggest that IGF-1 is primarily protective against initial demyelination and OL apoptosis, and as a result, IGF-1 treatment is only effective when given immediately following myelin insult. Consistent with this, a pilot clinical trial in seven MS patients with established disease onset found that treatment with recombinant human IGF-1 is ineffective [152]. Thus, despite the ability of IGF-1 to promote the survival of cells from the OL lineage and to enhance oligodendrogenesis under physiological conditions, results from IGF-1-based treatments in demyelinating disorders are mixed. IGF-1 may serve as a preventative treatment that decreases the initial disease burden but does not have persistent benefits. Clinical trials with MS patients early in disease onset and detailed studies of the in vivo mechanisms of IGF-1-induced oligodendrogenesis and remyelination are needed. Special attention should be paid to the experimental time points of IGF-1 treatment to determine whether IGF-1 is a viable preventative and/or therapeutic target for demyelinating conditions.

### 3.2. Insulin

Insulin is a metabolic hormone that classically regulates glucose homeostasis but also acts within the CNS in both an endocrine and paracrine fashion [153]. Insulin binds to the insulin receptor (IR), expression of which is detected in OLs [154], and can also bind to IGF1R, albeit with a lower affinity than IGF-1 [155]. While less is known about insulin’s IR-mediated effects on oligodendrogenesis, insulin likely increases OPC survival and differentiation via mechanisms akin to those of IGF-1 [33].

#### Implications for Disorders

Despite insulin’s ability to promote OL survival, treatment with insulin appears to be ineffective in demyelinating disorders. In both young and aged EAE rats, chronic treatment with IGF-1, but not insulin, ameliorates clinical severity scores [145]. Given that insulin requires considerably higher concentrations to promote OL survival compared to IGF-1 [156], this may suggest that insulin is not potent enough to protect against demyelinating insults.

Insulin’s effects on oligodendrogenesis may have implications for disorders characterized by significant disruptions in insulin signaling, such as type 1 and type 2 diabetes. Indeed, peripheral demyelination is a common diabetes-induced complication, and insulin signaling may contribute to myelin production in Schwann cells [157,158,159]. Disrupted insulin signaling may also affect myelination in the CNS. In cell culture, the absence of insulin decreases nuclear Olig1 levels in OPCs, which is necessary for differentiation of OPCs into mature OLs [160], and insulin contributes to OL survival [161,162]. Furthermore, patients with adult diabetes may present with abnormalities in white matter content that correlate with cognitive function, suggesting a potential link between blood insulin and white matter structure [163,164,165]. In middle-aged humans, both insulin resistance and insulin levels are associated with altered MRI-based estimates of myelin content [166]. However, in this study, there was region-related heterogeneity of these relationships; myelin content in the frontal and temporal lobes was positively correlated with insulin levels, while parieto-occipital myelin was negatively correlated with insulin levels. Such regional specificity has not been reported or thoroughly investigated in animal models of IGF1R upregulation. Future work could address this by investigating regional heterogeneity of the effects of insulin and IGF-1 signaling on brain myelination, as well as relating insulin action to myelination in animal models of obesity or diabetes. These data point to IGF-1 and insulin effects on oligodendrogenesis and myelin changes as a potential mechanism underlying the cognitive and neurological sequelae of diabetes. Overall, more research is needed to determine whether insulin acts on IR or IGF1R to alter myelination in diabetic humans and animals.

### 3.3. Prolactin

Prolactin is a peptide that promotes lactation and regulates diverse functions, including oligodendrogenesis [33]. While circulating prolactin is produced by the anterior pituitary, prolactin can also be produced locally in the brain [167,168,169,170,171]. Though effects of prolactin have not been studied across the entire OL lineage, prolactin increases OPC proliferation and differentiation under physiological conditions, suggesting it overall promotes oligodendrogenesis [172].

#### Implications for Disorders

Prolactin has garnered interest for its therapeutic potential in demyelinating disorders such as MS. This interest began with early observations that female MS patients show fewer relapses during the third trimester of pregnancy when prolactin and sex hormone levels are high [34]. Interestingly, prolactin plasma levels in female MS patients are positively correlated with white matter volume [173]. Given this relationship and the potential enhancement of oligodendrogenesis by prolactin in animal models [172], research has tested whether prolactin aids remyelination after myelin damage. Indeed, pregnancy is protective against LPC-induced demyelination in the mouse spinal cord, with pregnant mice displaying decreased lesion size and increased numbers of proliferative immature OLs at the injury site [172]. Although the necessity of prolactin signaling was not tested in this model, prolactin injections into virgin mice were sufficient to mimic this protective effect [172]. While this suggests positive effects of prolactin on OPC differentiation, the effects of prolactin injections on OL survival were not tested. Moreover, these experiments were conducted in female mice; the effects of prolactin on remyelination in male mice are not known.

While these results are promising, prolactin has known pro-inflammatory effects that could complicate its use as a treatment in disorders such as MS and antagonize its remyelination benefits [171]. In fact, lymphocytes produce prolactin, potentially serving as an autocrine signaling factor for these cells, and prolactin levels are elevated in a mouse model of EAE [171,174,175]. Indeed, in the EAE model, prolactin enhances lymphocyte proliferation in response to exogenous myelin proteins, and treating animals with prolactin has no effect on disease severity after EAE induction [176]. These findings may indicate that treating inflammatory-induced myelin damage with prolactin is not only ineffective but also potentially harmful and pro-inflammatory. Consistent with this, prolactin receptor knockout mice have a slight delay in EAE onset and develop full clinical severity [177]. However, coupling prolactin with interferon-β ameliorates clinical scores, suggesting that PRL treatment may be beneficial when prolactin’s actions on immune cells are inhibited [176]. Overall, these studies paint a complicated picture of prolactin’s role in oligodendrogenesis and remyelination after injury. Considerably more studies conducted both in vitro and in vivo will be necessary to parse prolactin’s separate actions on OLs and immune cells. Understanding the interaction of such effects will determine whether prolactin improves or hinders disease severity and disease-related oligodendrogenesis.

### 3.4. Melatonin

Melatonin is an indolamine neurohormone produced primarily by the pineal gland and, to a lesser extent, locally within the brain that regulates circadian rhythms and exhibits anti-inflammatory actions [178,179,180]. Both of the melatonin receptors are expressed by OLs [181]. Though the mechanisms remain unclear, melatonin increases NSC differentiation towards an OL fate, increases OL maturation both in vitro and in vivo, and enhances OL survival by inhibiting expression of the apoptotic factor caspase-3 [33].

#### Implications for Disorders

Few studies have examined the molecular effects of melatonin on oligodendrogenesis under physiological conditions; however, several have examined melatonin’s protective effects under demyelinating conditions. Such conditions include ischemia [182,183], stroke [184], and EAE [185,186]. These studies largely showed melatonin treatment mitigates the loss of MBP-positive fibers and mature OLs in regions such as the cingulum bundle, corpus callosum, and hippocampus [182,183,184,185]. In parallel with the amelioration of myelin loss were improvements in neurological disability scores [185,186]. In addition, three days of melatonin treatment rescued myelin density and partially restored the number of mature OLs in the cingulate and corpus callosum of neonatal rat pups subjected to uterine artery ligation [181]. These studies support the hypothesis that melatonin, acting either directly on OPCs and OLs, or indirectly via microglia and astrocytes, is protective against myelin damage. Notably, only one of these studies examined the effect of melatonin on oligodendrogenesis post-injury and found that melatonin treatment increased OPC proliferation [182]. The role of melatonin on OL survival, OPC differentiation, and OL remyelination is only beginning to be explored. Melatonin administered in the final seven days of cuprizone-induced demyelination in mice decreased markers of apoptosis in the corpus callosum; however, melatonin had no effect on remyelination [187]. This may suggest that melatonin’s effects are largely pro-survival, rather than pro-remyelination. Alternatively, longer treatment and/or observation times may be needed to observe the beneficial effects of melatonin. Interestingly, one study has suggested that melatonin administration in an adolescent rat EAE model exacerbates neurological disability scores [188]. However, whether melatonin’s protective effects are age-specific remains unresolved.

Many of the studies noted here also investigated the effects of melatonin on inflammation. Consistent with melatonin’s anti-inflammatory properties, melatonin treatment decreases pro-inflammatory cytokine levels in cuprizone-induced demyelination, EAE, and ischemia [182,184,185,186,189]. In addition, melatonin normalizes the numbers of reactive microglia and infiltrating lymphocytes in white matter regions and the spinal cord [184,185]. This may suggest that melatonin’s anti-inflammatory properties mediate its protective effects on myelination; however, none of these studies tested a causal link between these effects.

Interestingly, melatonin may exert pro-apoptotic effects on cancerous tissues, including oligodendrogliomas [190,191,192]. These effects appear to be limited to cancer cells [193], and melatonin broadly exhibits anti-apoptotic effects on healthy tissues or cells challenged by hypoxia or oxidative stress [180,194]. The mechanism of melatonin’s cancer cell-specific induction of apoptosis is poorly understood; however, one potential mechanism is melatonin’s regulation of mitochondrial membrane permeability. Under physiological or oxidative stress conditions, melatonin reduces levels of reactive oxygen species, activates anti-apoptotic signaling factors, and protects mitochondrial function [180,195,196]. However, these effects appear to be reversed in glioblastoma, with melatonin inducing reactive oxygen species and interfering with mitochondrial DNA transcription [192,197]; in addition, melatonin applied directly to mitochondria purified from the rat brain induces mitochondrial membrane permeability and mitochondrial release of pro-apoptotic factors and the myelin-associated enzyme, CNPase [198]. Though CNPase is often used as a marker of myelinating OLs, its contribution to mitochondrial function and its connection to OL survival and axonal maintenance are unclear. Overall, the effects of melatonin on mitochondrial function, particularly in OPCs and OLs in disease contexts, warrant further investigation and will be an interesting avenue for future research.

In sum, melatonin may be a promising candidate for the prevention of demyelination and the amelioration of disease severity; however, the mechanism of melatonin action remains unclear. Melatonin consistently attenuates myelin loss and reduces levels of pro-inflammatory cytokines and microglial reactivity; however, no study has demonstrated a causal link between these two parallel effects.

### 3.5. Thyroid Hormones (THs)

THs are tyrosine-based hormones that act on almost every cell type in the body to regulate CNS development, including oligodendrogenesis. [33,199,200,201]. These two hormones, the functionally active triiodothyronine (T3) and its precursor thyroxine, are essential for the development and differentiation of OLs [202]. THs’ pro-oligodendrogenesis effects are mediated in part through their action through the two forms of thyroid hormone receptors (TRs), TRα and TRꞵ. These TRs are nuclear receptors that bind either as homodimers or heterodimers to thyroid response elements in DNA to alter gene expression [203,204]. In addition, TRs dimerize with other nuclear receptors expressed in OLs [205], including retinoid X receptors, to exert effects on oligodendrogenesis [206,207,208].

#### Implications for Disorders

It has long been known that TH deficiency, in both humans and other animals, leads to impairments in myelin during development [209,210,211,212,213] and that treatment with TH can reverse myelination deficits if given within a critical developmental window [214,215]. In addition, due to its pro-oligodendrogenesis effects, THs have been studied as a potential treatment for demyelinating disorders [216]. In SCI in adult rodents, local delivery of T3 promotes new OL formation and increased myelination in vivo [217]. T3 also improves remyelination following cuprizone-induced demyelination [216,218,219,220]. For example, T3 increases numbers of both OLs and their precursors in the adult mouse brain following chronic demyelination [219]. Increases in TH-induced OPC differentiation following cuprizone injury are thought to be due to the TRꞵ, as TRꞵ was only faintly detected in the subventricular zone of demyelinated animals and receptor expression was upregulated following TH treatment [218]. TH may also improve re-myelination through upregulation of transcription factors like krüppel-like factor 9, a zinc finger transcription factor that aids in OPC differentiation [220].

In EAE models, TH again aids in remyelination [221,222,223,224,225]. Similar to effects observed during development, TH treatment inhibits NSC proliferation and upregulates markers of OPCs and mature OLs [222,223,224]. This increase in OL differentiation may, in part, be due to transcriptional upregulation of platelet-derived growth factor receptor alpha, another inducer of OL differentiation [224]. In addition to effects on OL differentiation and maturation, TH administration protects and repairs myelin sheaths, likely through enhancement of MBP protein expression [221,222,224]. Heterodimerization of TR with retinoid X receptors may play a role in remyelination, as 9-cis-retinoic acid promotes remyelination in concert with THs both in vitro, in cerebellar slice cultures after demyelination by LPC, and in vivo, in aged EAE rats after demyelination [226]. Protective effects of TH are not limited to rodent models; TH enhances remyelination in non-human primates with EAE and, importantly, improves clinical scores [225]. TH’s pro-remyelination effects may also be due to modulation of the immune system and immune factors such as inflammatory cytokines [227]. For example, T3 treatment reduces the number of pro-inflammatory IL-17-positive T-cells [228]. The interaction of THs and inflammatory markers will be an important area for future study. In fact, inflammation may reduce TH levels and that reduction in TH may play a causal role in MS pathology [229]. Overall, TH appears to be a promising avenue for treating demyelinating disorders, and clinical trials have recently begun testing the safety and efficacy of TH treatment for MS [230].

## 4. Future Directions

Broadly, hormonal actions on oligodendrogenesis represent an underexplored therapeutic avenue for myelin repair. Yet, as we have noted throughout this review, there are many unanswered questions. For example, while many studies have shown that hormones have neuroprotective and pro-remyelination effects in animal models of white matter damage, their mechanisms of action remain largely unclear. Understanding these mechanisms will be crucial for designing therapeutics that target specifically oligodendrogenesis and remyelination and minimize off-target effects in the CNS. In addition, many of these hormones both improve myelin loss and reduce inflammation. Thus, it remains to be determined if hormones act directly on the OL lineage to enhance remyelination or indirectly through interactions with the immune system and other factors.

In addition, few studies have addressed the complex interactions amongst the hormones themselves. For example, estradiol induces upregulation of PRs, and treatments with both estrogens and progesterone combined were more effective in restoring myelination [38,93,94]. In addition, prolactin ameliorates disease severity in EAE, but only when its modulation of immune cells is dampened [176]. Future work could continue to elucidate such pleiotropic effects of hormones in order to design combinatorial treatments for demyelinating disorders.

Further, we have little understanding of how hormones might affect oligodendrogenesis and myelin repair based on age. Indeed, the actions of several of the hormones discussed here change over the course of development and adulthood [33]. Thus, the effectiveness of hormonal strategies may depend on an individual’s age and their circulating levels of hormones, which differ in children, adolescents, and adults. Future studies in vivo should, therefore, test hormonal effects on oligodendrogenesis and remyelination across the lifespan. Similarly, there is a dearth of work testing hormonal effects on myelination in an aging context. Aging alone leads to white matter loss, and the effect is exacerbated in patients with AD [3,231,232,233]. While a handful of studies have demonstrated pro-remyelinating effects of hormones in an AD model [120,121], their mechanisms of action remain unclear and many of the classical hormones we described here have yet to be tested in AD models. Lastly, while we have detailed the effects of a range of different hormones on oligodendrogenesis and remyelination in the context of disease, there are many more hormones that might regulate oligodendrogenesis, including classical and non-classical hormones such as gut hormones and catecholamines. For example, in the mouse EAE model, the secretin hormone vasoactive intestinal peptide ameliorates disease severity, prevents demyelination, and prevents the death of mature OLs in the spinal cord [234]. Overall, the field has enormous potential, with numerous future directions.

## 5. Conclusions

Hormones across many classes exert protective and remyelinating effects on disorders characterized by myelin loss (Figure 1). Many of these hormones, including IGF-1, THs, and steroid hormones, act through both direct actions on OLs and indirect actions on the immune system. A better understanding of hormonal mechanisms and the circumstances under which they act, such as age and disease onset, will allow for more targeted therapeutics for disorders that lead to OL damage and demyelination.

## Data Availability

Not applicable.

## Abbreviations

AD	Alzheimer’s disease
AMPA	α-amino-3-hydroxy-5-methyl-4-isoxazolepropionic acid
AR	androgen receptor
CNPase	2’,3’-cyclic-nucleotide 3’-phosphodiesterase
CNS	central nervous system
Dex	dexamethasone
DHT	dihydrotestosterone
E2	17-β estradiol
EAE	experimental autoimmune encephalomyelitis
ER	estrogen receptor
GC	glucocorticoid
GR	glucocorticoid receptor
IGF-1	insulin-like growth factor-1
IGF1R	insulin-like growth factor-1 receptor
IR	insulin receptor
LPC	lysophosphatidylcholine
MBP	myelin basic protein
MP	methylprednisolone
MS	multiple sclerosis
NSC	neural stem cell
OL	oligodendrocyte
Olig1	oligodendrocyte transcription factor 1
OPC	oligodendrocyte precursor cell
OVX	ovariectomized
PR	progesterone receptor
PTSD	post-traumatic stress disorder
SCI	spinal cord injury
T3	triiodothyronine
TH	thyroid hormone
TR	thyroid hormone receptor
